# Plasma Exosome Profiling of Cancer Patients by a Next Generation Systems Biology Approach

**DOI:** 10.1038/srep42741

**Published:** 2017-02-20

**Authors:** Valeriy Domenyuk, Zhenyu Zhong, Adam Stark, Nianqing Xiao, Heather A. O’Neill, Xixi Wei, Jie Wang, Teresa T. Tinder, Sonal Tonapi, Janet Duncan, Tassilo Hornung, Andrew Hunter, Mark R. Miglarese, Joachim Schorr, David D. Halbert, John Quackenbush, George Poste, Donald A. Berry, Günter Mayer, Michael Famulok, David Spetzler

**Affiliations:** 1 Caris Life Sciences, 4610 South 44th Place, Phoenix, AZ 85040, USA; 2 Department of Biostatistics and Computational Biology, Dana-Farber Cancer Institute, 450 Brookline Avenue, Smith 822A, Boston, MA 02215, USA; 3 Department of Biostatistics, Harvard School of Public Health, 655 Huntington Ave, Boston, MA 0211, USA; 4 Complex Adaptive Systems Initiative, Arizona State University, 1475 N. Scottsdale Rd., Suite 361, Scottsdale, AZ 85257, USA; 5 Department of Biostatistics, University of Texas MD Anderson Cancer Center, Houston, Texas, USA; 6 LIMES Program Unit Chemical Biology & Medicinal Chemistry, c/o Kekulé Institut für Organische Chemie und Biochemie, University of Bonn, Gerhard-Domagk-Straße 1, 53121 Bonn, Germany; 7 Chemical Biology Max-Planck-Fellowship Group, Center of Advanced European Studies and Research (CAESAR), Ludwig-Erhard-Allee 2, 53175 Bonn, Germany

## Abstract

Technologies capable of characterizing the full breadth of cellular systems need to be able to measure millions of proteins, isoforms, and complexes simultaneously. We describe an approach that fulfils this criterion: Adaptive Dynamic Artificial Poly-ligand Targeting (ADAPT). ADAPT employs an enriched library of single-stranded oligodeoxynucleotides (ssODNs) to profile complex biological samples, thus achieving an unprecedented coverage of system-wide, native biomolecules. We used ADAPT as a highly specific profiling tool that distinguishes women with or without breast cancer based on circulating exosomes in their blood. To develop ADAPT, we enriched a library of ~10^11^ ssODNs for those associating with exosomes from breast cancer patients or controls. The resulting 10^6^ enriched ssODNs were then profiled against plasma from independent groups of healthy and breast cancer-positive women. ssODN-mediated affinity purification and mass spectrometry identified low-abundance exosome-associated proteins and protein complexes, some with known significance in both normal homeostasis and disease. Sequencing of the recovered ssODNs provided quantitative measures that were used to build highly accurate multi-analyte signatures for patient classification. Probing plasma from 500 subjects with a smaller subset of 2000 resynthesized ssODNs stratified healthy, breast biopsy-negative, and -positive women. An AUC of 0.73 was obtained when comparing healthy donors with biopsy-positive patients.

Extracellular vesicles (EV), which are secreted into circulation by many cell types, can provide a snapshot of cellular processes active in disease and healthy cells, allowing the exosomes in circulation to serve as sentinels of the health of an individual. In cancer, exosomes from neoplastic cells are involved in intercellular communication essential for several fundamental aspects of malignancy, including immune evasion^1^, angiogenesis^2^, and metastasis^3^,^4^. The molecular composition of exosomes correlates with the cell-of-origin^5^, and alterations in membrane components, luminal contents, and abundance^6^ of exosomes have been described in a variety of cancers^7^,^8^,^9^,^10^. Thus, exosomes may be an informative biological substrate, reflecting the dynamic alterations that can occur during tumour progression.

Libraries consisting of several trillion ssODNs encompass nearly infinite numbers of three-dimensional structures due to the vast complexity of DNA sequence space^11^,^12^,^13^. Selection/amplification schemes can be devised to scan this huge structural space for ssODNs that bind to simple or complex targets^14^,^15^. These qualifications enable parallel profiling of differences in molecular content across a wide range of biological sources without prior knowledge of binding partners^16^,^17^, but this potential has not been fully exploited to date.

Here we describe how libraries of ssODNs can be used to profile plasma exosomes from women with and without breast cancer. We introduce “Adaptive Dynamic Artificial Polyligand Targeting (ADAPT)”, a novel approach for monitoring differences in the molecular content of plasma exosomes in a massively parallel fashion and without prior knowledge of the targets.

## Results and Discussion

**Figure 1:**
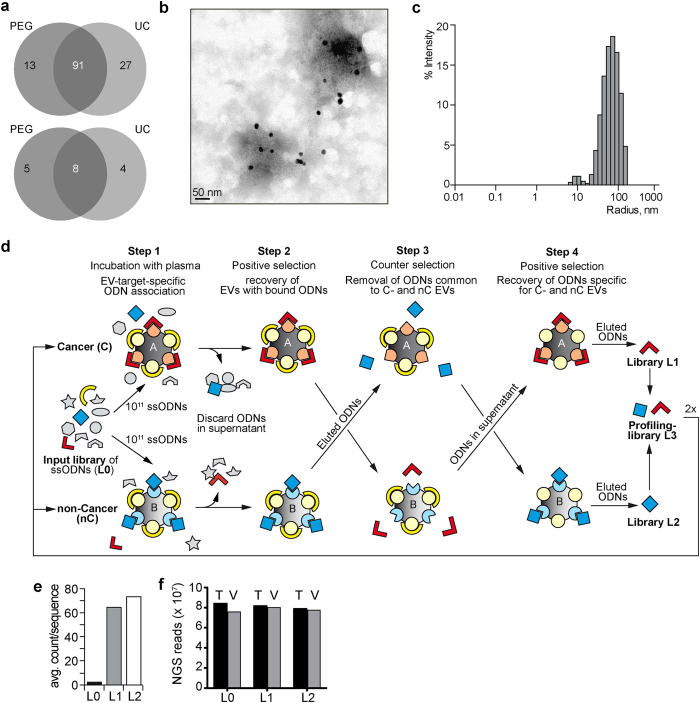
Generation of Profiling Library for ADAPT. (**a**) Venn diagram showing the overlap between exosome-associated (top) and non-exosome-associated (bottom) proteins identified in PEG- or UC-precipitated plasma pellets. (**b**) TEM images of PEG precipitated exosomes (EV) visualized by anti-CD9 antibody coupled gold-nanoparticles (black spheres). (**c**) Dynamic light scattering (DLS) analysis of EV sizes distribution isolated by PEG precipitation. The signal decay curve as well as DLS of controls (UC purified plasma exosomes and exosome-free protein solution) are shown in Supplementary Figure 1g. (**d**) Library enrichment principle: a high-diversity molecule library (~10^11^ representatives) is contacted with blood plasma from biopsy-positive (Cancer, C) and, in parallel, with plasma from biopsy negative (non-Cancer, nC) individuals; in the 2^nd^ step non-bound ssODNs are removed with supernatant and bound molecules are collected; in the 3^rd^ step, ssODNs recovered from C are incubated with nC (and *vice versa*) and non-binders are removed with pellets; the 4^th^ step is another binding to positive target as outlined in steps 1^st^ and 2^nd^. This enrichment process was repeated three times with PCR in between: in the first iteration, UC was used to recover EVs; in the following two iterations, PEG-precipitation was used for EV recovery. A total of 119 biopsy-positive (n = 59), biopsy-negative (n = 30), and self-declared normal (n = 30) patient samples were used in the first round of L3 enrichment, while only the cancer and non-cancer samples were used in the subsequent rounds. This enrichment process delivers two separate lower-diversity libraries (~10^6^ representatives) enriched to features (targets) present in samples *A* and *B*, respectively. (**e,f**) Quality and population assessment of the starting library (L0) and the respective libraries L1 and L2 obtained from (**d**). NGS sequence depth was similar for L0, L1 and L2 (**f**, grey bars: valid reads V, black bars: total reads T), whereas the number of copies per sequence was significantly increased in L1 and L2 (**e**), while the total number of unique sequences was reduced (Supplementary Fig.1i).

ADAPT relies on sample fractionation to identify and characterize specific subpopulations of macromolecules and complexes in blood plasma, including those residing on the surface of exosomes. We used polyethylene glycol (PEG) precipitation (PPT)^18^ and ultracentrifugation (UC) to recover exosomes from blood plasma samples of healthy donors and analysed the protein content by LC-MS/MS (Supplementary Fig. 1a). A total of 131 exosome-associated proteins^19^ (Supplementary Table S1) were identified from PPT and UC pellets by LC-MS/MS analysis (Fig. 1a, upper panel). Among them, 13 were specific to PPT, and 27 to UC. Identified proteins comprise integral, peripheral, and lipid-anchored membrane proteins^20^, but also proteins with unknown membrane interaction (Supplementary Fig. 1b–e). In addition, PPT and UC identified 17 non-exosomal components, 5 specific to PPT, and 4 to UC (Fig. 1a, lower panel).

Transmission electron microscopy (TEM) was used to analyse the material collected by PPT, and exosome-like morphologies comparable to exosomes isolated by UC^21^,^22^ were confirmed. TEM-imaging with an *anti*-CD9 antibody and 10 nm colloidal immunogold (Fig. 1b), and Western blotting (Supplementary Fig. 1f) confirmed the presence of the canonical exosomal marker protein CD9 on the surface of PPT-derived exosomes. Dynamic light scattering (DLS) revealed 20–200 nm particles (Fig. 1c) with a robust signal decay curve (Supplementary Fig. 1g, left panel) confirming the reliability of the DLS measurements. The particle size is consistent with the size observed for UC-precipitated exosomes (Supplementary Fig. 1g, middle panel) versus the “no-plasma”-control (Supplementary Fig. 1g, right panel).

As the initial step in developing ADAPT, a trained library of randomized ssDNA sequences (called the profiling library L3) was generated from an unselected random starting library (called L0; Fig. 1d). Four different enrichment schemes were used to generate ssODN libraries specific for exosomes (Supplementary Table S2). Enrichment under the highest stringency was performed as follows: an aliquot of 10^11^ sequences L0 (Supplementary Fig. 1h) was either incubated with pooled blood plasma from 59 patients with positive breast cancer biopsy (Fig. 1d, Cancer; Supplementary Table S3), or with pooled blood plasma from 30 biopsy-negative patients and 30 self-declared healthy women (Fig. 1d, Non-cancer; Supplementary Table S4). All blood samples were collected prior to breast biopsy. Exosomes were UC-purified from both samples (Fig. 1d, step 2) and exosome-associated ssODNs were recovered from the precipitate. To enrich for ssODNs specific to each sample cohort, we used a counter-selection step whereby each enriched library was incubated with plasma from the other cohort (Fig. 1d, step 3) and the ssODNs contained in the exosomal fraction were discarded under the assumption that these would be binding to common elements not specific to disease. We then performed a second positive selection (Fig. 1d, step 4) by using the ssODNs contained in the respective supernatants from step 3 as input to repeat step 2. Exosome-associated ssODNs were recovered, representing enriched libraries, called L1 for positive biopsy patients, and L2 for the control cohort. Finally, L1 and L2 were amplified by PCR, converted to ssDNA, and mixed to yield library L3. This enrichment scheme was repeated twice using L3 as the input and substituting UC for PPT. The three rounds of selection reduced the complexity of the ssODN library to ~10^6^ different sequences and enriched for those that are associated with targets characteristic for exosome-populations from both sources, i.e. ssODNs that associate with molecules preferentially expressed in each cohort.

The libraries L1 and L2 were then characterized using next-generation-sequencing (NGS; see Methods section “enrichment of aptamer library” and Supplementary Table S5 for NGS general statistics and QC metrics data). NGS of the initial L0 library shows that the vast majority of sequences exist in low copy numbers (Fig. 1e and Supplementary Fig. 1h), while the final L1 and L2 libraries show significantly higher average counts per sequence (Fig. 1e) and reduced complexity (Supplementary Fig. 1i) with unaltered total valid reads (Fig. 1f). The increase in average copy number and the reduction of unique sequences are consistent with a successful enrichment process.

**Figure 2:**
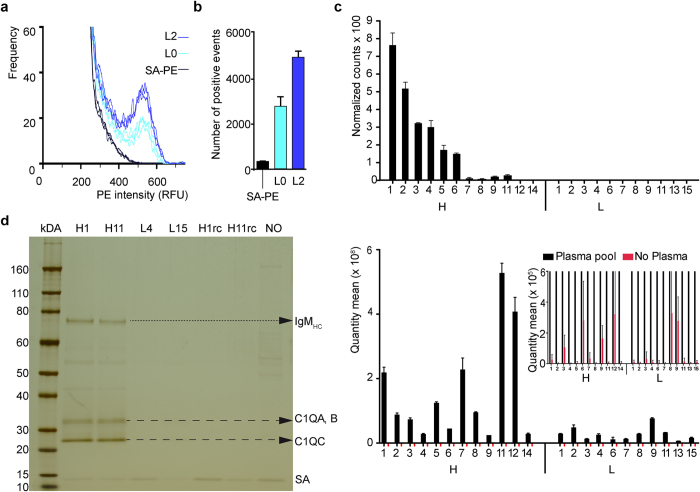
Enriched ssODNs interact with the surface of exosomes. (**a**) The enriched 5′-biotinylated ssODN library L2 (blue curves) reveals enhanced binding, measured by flow-cytometry, to UC isolated exosomes, compared to the 5′-biotinylated starting library L0 (cyan curves), or absence of ssODNs (w/o; black curves) using SA-PE as a staining agent, and gated only on double-positive CFSE+DiD+ events (Supplementary Fig. 2). The curves show the distribution of relative fluorescence intensities for observed events. Each curve represents an independent binding experiment (n = 3). (**b**) Total number of positive SA-PE staining events from (**a**) (number of events >RFU 400) of biotinylated L0 (cyan), and biotinylated L2 (blue), compared to absence of ssODNs (w/o; black), in exosome binding. In each binding reaction 12 nM of each library was used. (**c**) Post-ADAPT quantification of binding of individual aptamers as part of the library and individually as illustrated in Supplementary Fig. S3. The top panel shows 23 representative individual aptamers, selected either with high “H” or low “L” normalized counts from L3 NGS data after binding pooled plasma from breast biopsy negative donors. There is at least a 5-fold difference between counts of H and L ssODNs. These 23 sequences were re-synthesized and tested individually in the same binding assay, but in equal concentrations, unlike their original representation in L3. PEG-precipitated aptamer/plasma complexes were directly subjected to qPCR (bottom panel). Inlay: Magnification of qPCR results of ssODNs incubated with PBS instead of plasma (red). (**d**) Silver-stained reducing SDS-PA gel of pulled down proteins from PPT plasma with the indicated ssODNs (H1, H11, L4, L15), immobilized on streptavidin magnetic beads, and the control without ssODN (NO). Dashed arrows indicate pulled down proteins C1QA, C1QB, and C1QC. The dotted arrow indicates the heavy chain of IgM (IgM_HC_). SA: streptavidin. RC: reverse complement sequence.

To confirm that the enriched ssODNs interact with the surface of exosomes, we performed flow cytometry analysis of 5′-biotinylated libraries L2 and L0, respectively, using UC-purified exosomes from pooled plasma of a cohort of self-declared healthy human donors (Fig. 2a). Confirmation and target identification experiments were only performed on samples from healthy individuals due to the large sample volumes (>195 mL) required and the limited amount of plasma available from cancer patients. Exosomes were purified by UC and triple-stained with a lipid-intercalating dye (DiD) to confirm the presence of a lipid bilayer, an esterase-sensitive dye (CFSE) to confirm the presence of a lumen (Supplementary Fig. 2), and streptavidin-phycoerythrin (SA-PE) to confirm the presence of biotinylated ADAPT-ssODNs on the surface of exosomes. We found a significant increase in frequency of positive exosome-ssODN binding events with library L2 as compared to the starting library L0 (Fig. 2a,b). In the absence of ssODNs, no increase in the number of positive events was detected (Fig. 2a,b, SA-PE). Moreover, to verify whether L3 is suitable for the potential profiling of up- and down-regulated markers in plasma samples, we probed individual and pooled plasma samples (Supplementary Table S5). NGS based normalized counts can reflect differential expression of the molecular targets of the ssODN, recovered from the binding test as illustrated in Supplementary Figure 3 and Fig. 2c top panel (see also Supplementary Table S6).

To test the individual performance of ssODNs from L3, we synthesized 12 ssODNs that were at high abundance (termed “H”) and 11 that were at low abundance (termed “L”) (Supplementary Table S6). We chose H- and L-sequences based on at least 5-fold change in normalized counts from the NGS data of L3-probed pooled plasma from breast biopsy negative donors (Supplementary Table S6). These sequences were re-synthesized and tested individually against the same plasma pool, but in equal concentrations (Supplementary Fig. 3), using qPCR as readout. NGS quantified recovery of each sequence in L3 (Fig. 2c, top panel) can be directly compared with copies of these sequences, quantified by qPCR (Fig. 2c, bottom panel). The workflow of this experiment is described in Supplementary Figure 3. Individual binding tests confirmed that H-sequences generally have higher copy numbers than the L-sequences (Fig. 2c, bottom panel), consistent with our selection rationale based on NGS-derived copy numbers. Furthermore, L11 for example has greater relative recovery compared to NGS, where it was under-represented in L3. Increasing concentration however did not allow L-sequences to demonstrate comparable level of binding. Consequently, the quantity of the recovered ssODN indicates the abundance of the target. Sequences H4, H9 and H14 showed recovery similar to L-sequences; they likely represent non-specific binders. When the experiment was repeated in absence of plasma the recovery of ssODNs was barely above background (Fig. 2c inlay, red columns).

We next sought to identify target proteins of individual sequences by pull down experiments. Two high-recovery (H1, H11) and two low-recovery ssODNs (L4, L15) were immobilized on streptavidin magnetic beads and incubated with PEG-precipitated plasma from the pool of healthy donors. Proteins that remained bound after washing were eluted and analysed by denaturing PAGE (Fig. 2d). Specific bands seen in the H1 and H11 pull-down experiments (Fig. 2d, dashed arrows) were excised and analysed by LC-MS/MS. This analysis revealed the plasma- and exosome-associated^23^,^24^ protein C1Q as the target of sequences H1 and H11. Reverse complement versions of H1 and H11 (H1rc, H11rc) did not pull down C1Q. We also found that H1 and H11 co-precipitated IgM with C1Q (Fig. 2d, dotted arrow), consistent with previous observations where IgM was found to remain complexed with C1Q in sera that were precipitated with PEG^25^.

**Figure 3:**
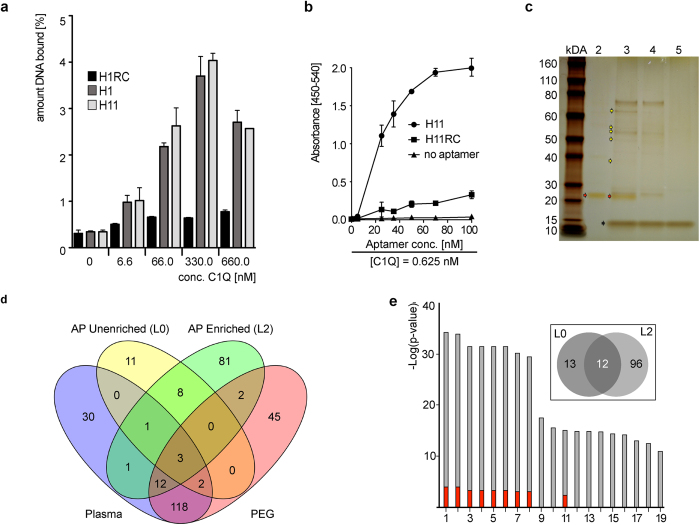
Oligonucleotides identified by ADAPT reveal aptamer-like characteristics. (**a**) Filter retention analysis of C1Q-binding by the ssODNs H1 (dark grey columns) and H11 (pale grey columns) at indicated C1Q concentrations. As a control, the reverse complement of H1, H1RC, was used (black columns). (**b**) ELONA analysis of C1Q-binding by the ssODNs H11 (circles) at indicated concentrations and a fixed C1Q concentration (0.625 nM). As a control, the reverse complement of H11, H11RC, was used (squares). “No aptamer” control (triangles) shows low background binding of detector Streptavidin-HRP. H11 specifically binds C1Q (estimated K_D_ around 40 nM). (**c**) PAGE analysis of PEG-precipitated and ssODN-associated proteins pulled-down with L2. Lane 1: Molecular weight marker; lane 2: Input library L2; lane 3: Fraction pulled down by biotinylated L2; lane 4: Fraction pulled down by non-biotinylated L2; lane 5: Fraction found in the absence of DNA library; red arrows indicate the ssODN library; yellow arrows indicate protein bands cut out and analysed by LC-MS/MS; black arrows indicate streptavidin monomers leaking from beads. (**d**) Four-way Venn diagram of proteins detected by LC-MS/MS from Unfractionated plasma, PEG-precipitated plasma, PEG-precipitated plasma in presence of L0 or L2, respectively, purified by streptavidin magnetic beads (background-subtracted; i.e. biotinylated minus non-biotinylated libraries). (**e**) Gene ontology (GO) cellular component enrichment analysis of the subset of proteins associated with L0 (red bars) and L2 (grey bars) that show a p value of at least 6 × 10^−12^. Proteins listed are: 1 extracellular region part, 2 extracellular region, 3 extracellular exosome, 4 extracellular membrane-bounded organelle, 5 extracellular organelle, 6 extracellular vesicle, 7 membrane-bounded vesicle, 8 vesicle, 9 organelle, 10 membrane-bounded organelle, 11 extracellular space, 12 focal adhesion, 13 cell-substrate adherence junction, 14 cell-substrate junction, 15 adherence junction, 16 anchoring junction, 17 cellular component, 18 cell junction, 19 blood micro particle. Inset: 108 proteins pulled down by L2 (inset, 96 + 12), cut from the gel shown in (**c**), and analysed by LC-MS/MS, 13 proteins unique to background-subtracted L0 (inset, 13), and 12 overlapping proteins (inset, 12).

To further validate the interaction of aptamer H1 and H11 with C1Q, we performed a filter retention assay (Fig. 3a). Radioactively labelled H1 and H11 were incubated with increasing concentrations of purified C1Q and subsequently passed through a nitrocellulose membrane. The amount of ssODN retained on membrane-immobilized C1Q was then quantified by autoradiography. These experiments demonstrate that H1 and H11 exhibit concentration dependent binding to C1Q, whereas H11RC did not. Similar results were obtained with H11 and H11rc in an Enzyme-Linked Oligonucleotide Assay (ELONA; Fig. 3b). This binding behaviour indicates that H1 and H11 identified by ADAPT specifically interact with C1Q with high affinity. These results underline aptamer-like interaction properties of the ssODNs enriched by ADAPT and show that exosome-associated proteins are targets of ssODN enrichment by ADAPT.

To fully exploit the massively multiplex nature of L2, we used the entire library to characterize the biological system, significantly increasing the robustness with which we can characterize molecules and molecular complexes occurring on the surface of exosomes. To test whether the simultaneous identification of multiple putative protein-targets for ssODNs contained in L2 is possible, we performed a L0- and L2-mediated affinity pull-down assay using pooled plasma from healthy donors stratified by denaturing and reducing PAGE (Fig. 3c). Individual bands (MW 30–80 kDa) were excised from the gel, digested and analysed by LC-MS/MS. As a control, the background was established by analysing PPT and neat plasma. The overlap in the number of unique proteins detected in these preparations is shown in the Venn-diagram in Fig. 3d and listed in Supplementary Table S7. Remarkably, 81 unique proteins were pulled down by L2 that were not detectable in the PPT and neat plasma, indicating that these low abundant potentially informative proteins became enriched due to their interaction with L2 ssODNs. Notably, some of the L2 enriched proteins are known for their roles in tumour suppression (Supplementary Table S7; Gene ID: *HIST1H2BK, MORC, AHNAK, TRIM29, ANXA1, ANXA2P2, ZDBF2, 1433S, LGALS7, SFPQ*), while some proteins known to regulate p53 IRES mRNA were also detected (Supplementary Table S7; Gene ID: *SFPQ, hnRNPA1/A2, VCP, ANXA2P2, HSP90AA1/AB1, eIF3,* and *RPS19*). The absence of these proteins in the L0-mediated pull-down demonstrates that the library L2 is enriched for ssODNs that bind to specific proteins and protein complexes, some with known relevance in both normal homeostasis and disease.

The subset of proteins associated with L0 and L2, respectively, was then analyzed by gene ontology (GO) cellular component enrichment^26^ (Fig. 3e and Supplementary Tables S8 [L0], 9 [L2]). Figure 3e shows the classifications of identified proteins by L2 (grey) and L0 (red) pull-down that have a *p*-value smaller than 6 × 10^−12^. L2 is able to bind and pull-down specific proteins. Moreover, GO categories “Molecular Function” (Supplementary Table S10) and “Biological Process” (Supplementary Table S11) identified proteins enriched for specific GO terms in L2 pull-down samples not detectable in the L0 pull-down. Notably, GO Molecular Function Enrichment Analysis (Supplementary Table S10) revealed significant enrichment of nucleic acid binding proteins (p < 10^−9^). The functional relation to exosomes of the 108 proteins pulled down by L2 and the 25 proteins pulled down by L0 (Fig. 3e, inlay) is further specified in Supplementary Fig. 4. These data suggest that the ssODNs act as aptamers, binding specific exosome-associated proteins and protein complexes.

MS-based analysis confirmed that the enriched library binds significantly more targets, compared to the parental, unenriched library. As the next step, NGS-derived counts of ssODNs recovered in the probing of the plasma samples were used in a massively parallel manner to develop multi-analyte signatures for classifying patients that belong to specific populations without precise information on the actual underlying ssODN-target pairs. Thus, valuable quantitative information that allows the differentiation of patient samples can be generated by NGS analyses of the recovered ssODNs from individual patient samples and by comparing the relative copy number of individual sequences. This new approach was termed ADAPT and here we demonstrate its utility on breast cancer patients compared to biopsy negative and normal donors.

**Figure 4:**
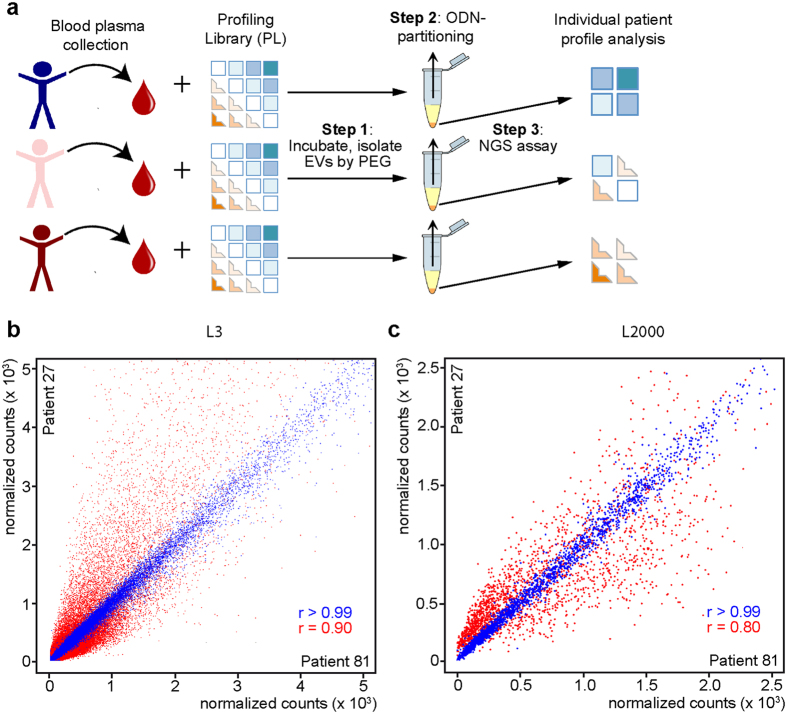
Adaptive Dynamic Artificial Poly-ligand Targeting (ADAPT). (**a**) Workflow: In the 1^st^ step plasma samples of breast cancer patients are incubated with the profiling library L3 (obtained in the enrichment outlined in Fig. 1d). After incubation EVs are precipitated and supernatant is removed (step 2). EV-associated oligonucleotides are subjected to next generation sequencing (step 3, NGS) to obtain a patient individual profile. (**b**) Correlation between technical replicates and biological samples for L3. The scatter plot shows the distribution of normalized counts of aptamers recovered from ADAPT with the plasma aliquots of the same patient (blue dots, r > 0.99; intra sample: patient 27) or plasma samples from different patients (red dots, r = 0.90). Every individual dot represents a unique sequence with the count of that sequence corresponding to representation in different samples or technical replicates. (**c**) Correlation between technical replicates and biological samples for L2000. Scatter plot shows distribution of normalized counts of aptamers recovered from ADAPT with the plasma aliquots of the same patient (blue dots, r > 0.99; intra sample: patient 27) or plasma samples from different patients (red dots, r = 0.80). Every individual dot represents a unique sequence with the count of that sequence corresponding to representation in different samples or technical replicates.

The general workflow of ADAPT used to profile exosomes/proteins from individual patients is illustrated in Fig. 4a. L3 was incubated with plasma samples from individuals, followed by PPT (Fig. 4a, step 1). ssODNs were recovered (Fig. 4a, step 2) and quantified using NGS to create patient-specific ssODN profiles (Fig. 4a, step 3).

To assess the technical reproducibility and complexity of biological information mirrored in the ADAPT profile, normalized counts of every unique sequence were compared within (Fig. 4b, Supplementary Fig. 5, blue) and between patients (Fig. 4b, Supplementary Fig. 5, red) for a library composed of 10^6^ unique ssODN. In all cases, sequence distributions were highly correlated between technical replicates (blue; *R*^2^ > 0.99). In contrast, sequence distributions between different individuals are highly variable (red; *R*^2^ = 0.74–0.90). Thus, the assay quantifies the number of binding events for 10^6^ ssODNs per patient, where changes in normalized counts of ssODNs can identify differences between patients and populations. These results demonstrate that L3 contains ssODNs capable of reflecting the biological differences between individual patient plasma samples. Thus, ADAPT might reveal disease related patient profiles.

L3 is an enzymatically, *in vitro* selected ssODN library that contains ~10^6^ molecules each present at a different concentration. To improve the efficiency of patient profiling and to gain control over library composition and concentrations of individual ssODNs, we developed a synthetic library able to differentiate cancer patients from controls. In this way, 2000 ssODNs (Supplementary Table S2) were selected from 4 different enrichment schemes (Supplementary Table S2) following the general outline depicted in Fig. 1d on different patient cohorts (Supplementary Table S12). Individual ssODNs were chosen from these libraries based upon their significance in t-test and fold change comparing either pools of patient samples (cancer vs. controls) or in testing a small cohort of patients (Supplementary Table S13). Each of the 2000 ssODNs were synthesized and then mixed at equimolar concentrations to form library L_2000_. The biological and technical diversity for L_2000_ showed the same relation as L3 on the same patients shown in Fig. 4b (Fig. 4c and Supplementary Fig. 6), thus confirming that the information contained in L3 was preserved in L_2000_.

**Figure 5:**
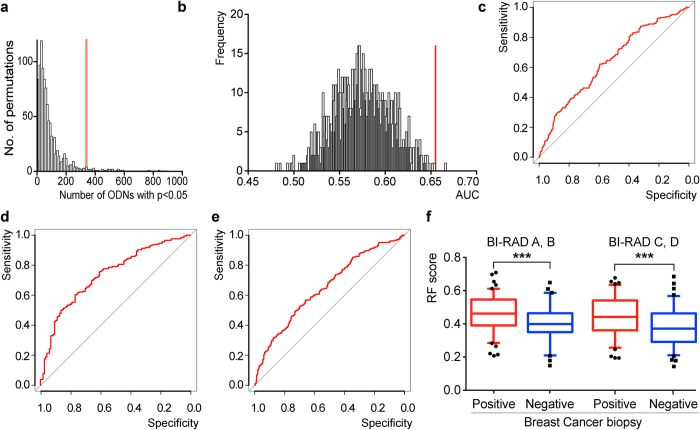
Assessment of ADAPT profiles of 500 clinical samples suggests sample type discrimination. (**a**) F-test for cancer versus non-cancer versus negative on original (red line) and permutated (columns) datasets. The number of ssODNs that distinguish breast cancer biopsy positive from negative and self-declared normal samples is significantly higher then can be achieved randomly in 1000 permutations of the original dataset. This shows that the selected ssODNs from L_2000_ reveal information that distinguishes the analysed cancer population from the other, confirming the applicability of the enrichment strategy and the proper compilation of the small profiling library L_2000_. (**b**) OOB AUC from the entire set of 500 clinical samples and permutation analysis of its reliability. AUC in the original dataset is 0.66 (red line), which is significantly higher compared to the majority of 1000 permutations (p = 0.002; columns). (**c**) ROC curve for cancer versus control samples with AUC = 0.66; *p* = 0.002. (**d**) ROC curve for cancer versus healthy donors with AUC = 0.73; *p* = 0.001. (**e**) ROC curve for cancer versus biopsy negative with AUC = 0.64; *p* = 0.024. (**f)** RF based probability of a cancer incident in both biopsy positive and negative sample types and corresponding radiographic Bi-RAD C&D scores suggesting that ADAPT reliably differentiates cancer from control groups even when using samples from women with dense breast tissue (BI-RAD A&B and C&D Cancer/Control p < 0.001).

To evaluate the utility of the L_2000_ library in providing differences of patient-specific information, 500 patients [206 cancer patients (Supplementary Table S14), 177 breast biopsy negative (Supplementary Table S15), 117 self-declared healthy] were profiled and differences in recovered copy number measured by NGS compared to ascertain the ability of ADAPT to stratify disease-related patient populations. Given the large number of ssODN that can be tested simultaneously, there is significant risk of false discovery of ssODNs that appear to be informative when they are not. Thus, to mitigate the risk of false discovery, we used permutation testing to check whether the library truly contained information specific to each patient group. Briefly, the number of ssODNs with p < 0.05 was determined using an ANOVA F-test across the 500 samples between the biopsy positive, biopsy negative and healthy groups. This value was then compared to the number of ssODNs found when the samples were re-grouped randomly and independently of their cancer status. The distribution of the number of significant ODNs in the permuted and cancer-control groups is shown in Fig. 5a. The observed difference (p = 0.026) in the performance of random groups compared to the original cancer-control group shows that there is a significant number of selected ssODNs from L_2000_ that reveal information differentiating the biopsy positive, biopsy negative, and healthy groups, confirming that the enrichment strategy and the ssODN selection logic produce informative libraries.

We next built a classifier using a random forest (RF) algorithm to assess whether the ssODNs can identify the different populations in the study. Feature selection was based on the significance of a particular ssODN between cancers and controls for all 500 samples using t-test (p < 0.05), Wilcoxon rank-sum test (p < 0.05), Kolmogorov-Smirnov test (p < 0.05) or fold change (>1.2) followed by RF algorithm generation; 289 ssODNs were included in the RF algorithm and used across the 1000 trees. Area under the curve (AUC) values based on prediction of out-of-bag (OOB) samples (316 training, 184 test) was employed to assess the combined utility of the ssODNs to differentiate the cohort of cancer patients from the control population (Fig. 5b, red line, AUC = 0.66). The specificity of L_2000_ to differentiate cancer from control relative to random groupings of the patients tested was evaluated by permutation analysis (Fig. 5b grey bars, permutation p = 0.002). Only 0.2% of randomly permutated datasets exceeded the performance of the original set. This result indicates that the information contained in the ssODNs enriched on a distinct subset of patients extends to a cohort of 500 patients and is capturing information specific to the cancer status of the individuals tested. The discriminating power of the ssODNs was evaluated using RF for cancer vs. biopsy negative, cancer vs. healthy, and cancer vs. all controls. Specific AUC values for each comparison were 0.64, 0.73 and 0.66 (Fig. 5c–e) with permutation *p*-values of 0.024, 0.001 and 0.002.

Notably, there is a significant increase in the ability of L_2000_ to differentiate healthy donors from cancer patients relative to biopsy negative patients. This suggests that the L_2000_ is measuring components related to abnormalities within the breast. Since there was no detectable difference in data due to Bi-RAD grouping, the method is independent of breast tissue density (Fig. 5f). These results suggest that refinement of the diagnostic performance could lead to a valuable tool that will aid in the diagnosis of breast cancer when mammography is uninformative.

## Conclusion

Our study demonstrates that the binding profile of diverse ssODN libraries, enriched by “plasma-SELEX”, can be used to identify breast cancer and to reveal differences in tumour biology. In principle, the ADAPT platform offers a novel high feature set tool for unbiased precision profiling of molecular biosignatures in both healthy and disease states. The signature provided by ADAPT profiling of exosomes and other plasma content also offers a robust method for identifying and characterizing individual molecules as well as multi-molecular complexes that comprise comprehensive interactomes associated with health and disease which may lead to new therapeutic targets and/or diagnostic markers.

ADAPT-based profiling of plasma exosomes combines many advantages of other proteome-wide profiling tools such as cell^16^- or tissue-SELEX^17^, and aptamer^27^ or antibody^28^,^29^ arrays, while avoiding certain disadvantages: i) ADAPT constitutes a minimally invasive platform that should be compatible with liquid biopsies; ii) exosome-based screening circumvents potential limitations associated with cell-based SELEX^16^,^30^,^31^, in particular the “dead-cell problem” that might hamper efficient aptamer enrichment^32^, or the fact that most reports on cell-SELEX employ cultured tumor cell lines that often, after many passages, do not reflect the original tumor composition anymore; iii) no prior knowledge on the nature of informative targets is required; iv) the ADAPT-based retrieval of information contained in patient samples is not limited to a particular disease, proteins or protein complexes but can in principle include other classes of molecules, like miRNAs, glycosylation-patterns or nucleic acid/protein complexes that may carry information relevant for meaningful patient stratification; v) ADAPT achieves the enrichment and identification of low abundance targets.

Few aptamer-based molecular profiling approaches have been published to date, one of the most prominent being the SOMAscan platform that employs a defined set of more than 1000 chemically modified aptamers, each of which was selected to bind a specific known target^33^. SOMAscan has been applied for the proteomic analysis of exosomes from the prostate cancer cell line DU145, and more than 100 proteins were identified that were enriched in exosomes relative to cells^27^. Thus, SOMAscan, and likewise antibody/ELISA-based platforms^34^, are used to validate whether or not any of the covered targets is associated with a specific biological condition. These approaches thus can be considered a “reverse chemical profiling” approach [in analogy to reverse (chemical) genetics]^35^. ADAPT differs from these approaches in that pre-trained without any prior knowledge about the target ssODN libraries are used for system-wide probing of plasma samples from hundreds of individuals in parallel. ADAPT thus is analogous to a “forward chemical profiling” approach that intrinsically allows for the identification of sets of novel targets. ADAPT-ssODNs can subsequently be used to identify targets that are associated with a particular biological condition (here: cancer vs. non-cancer). Our exemplary identification of C1Q as a protein-target associated with an individual sequence shows that target recognition can occur via aptameric binding mechanisms. Whether aptameric protein-recognition represents the only mechanism by which an ssODN becomes enriched during ADAPT-library training is presently unknown, but in principle, ADAPT should also allow for the enrichment of ODNs that target biomolecules other than proteins, such as protein-bound RNAs, posttranslational modifications, or miRNAs. “Reverse chemical profiling” platforms that are aptamer-, SOMAmer- or antibody-based, however, are currently restricted to exactly the protein-targets that they cover.

While ADAPT in its current form still requires further refinement to bring it to a clinical standard, we envision that with more extensive training, libraries of increasing quality, information content and greater AUC-values will become available that will provide a useful tool for the massively parallel analysis of complex interactome networks of biomolecules in their native state in healthy and diseased conditions.

## Methods

### Patients

Bio-specimens utilized in this experiment were obtained under an IRB-approved Bio-repository Protocol (see the list of IRB committees below). All subjects were consented with an IRB approved consent form and per 21 CFR 50.20 guidelines. Informed consent was obtained from all subjects. All experimental procedures in this publication were reviewed by the Western Institutional Review Board (WIRB) and deemed to fall under IRB Exemption per 45 CFR 46.101(b)(4). All experiments were approved by WIRB and performed in accordance with the Code of Federal guidelines and regulations.

List of committees: Western IRB (Sponsor’s Central IRB). Additional Local IRBs consist of: CAMC/WVU Charleston IRB; Phoebe Putney Memorial Hospital (PPMH) IRB; MedCentral IRB; LVHN IRB; Margaret R. Pardee Memorial Hospital IRB; Community Healthcare System Central IRB; Hartford Healthcare IRB; Renown Regional Medical Center IRB; John Muir Health IRB; Jupiter Medical Center IRB; Middlesex Hospital IRB; Orlando Health/Orlando; Regional Healthcare System IRB; MPM-SAH IRB; St. Luke’s Hospital and Health Network IRB; CCHC IRB; MCW/FH IRB; Bon Secours Richmond Health System IRB; Roper-St. Francis Healthcare IRB; CPHS; Greater Glasgow & Clyde Health Board.

Demographics and clinical characteristics of the patients used in this study can be found in the Supplementary Tables S3, S4, S12, S13, S14 and S15.

### Specimen characteristics

Blood plasma was collected at multiple collection sites using standardized protocol and the centrifuges provided by Caris Life Sciences. Blood was collected using standard venipuncture technique into four purple-top EDTA tubes, gently inverting each tube 8–10 times after collection. After sitting upright at room temperature for at least 30 min, but no longer than 6 hrs, the tubes were spun in the Labofuge 200 for 10 min at 5300 RPMS with proper balance. Tubes were carefully removed from centrifuge and to avoid sudden movements in an effort to ensure blood separation is maintained. Plasma was collected (3 × 1.2 ml) from a tubes in collection order and transferred into the cryovials in sequential order (a, b, c), utilizing the pipette working from the top layer of plasma down towards the Buffy Coat using great care not to disrupt the Buffy Coat. All cryovials were immediately placed into the −20 °C freezer. Total collected is 12 cryovials (3 fractions from 4 tubes). Samples were shipped using the Dry Ice Kit after at least 24 hours in freezer, in the sealed biohazard bag. Samples were excluded if haemolysis score exceeded 100 mg/dl of hemoglobin concentration (http://www.mayomedicallaboratories.com/articles/communique/2008/12.html).

Samples combinations and volume for the following assays:

#### Enrichment

1.2 ml × 59/60 Breast Cancer Biopsy Positive/Negative samples in the pool. Particular round of enrichment requires 300 μl of pooled plasma.

#### Profiling

200 μl of plasma from individual 500 samples in 3 technical replicates.

### ssODN library design and production

**Figure 6:**
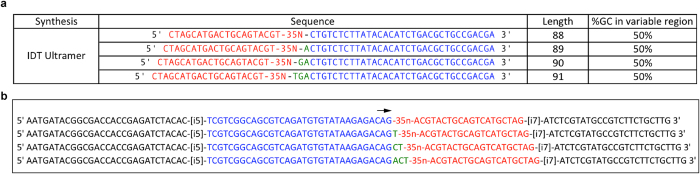
Design of the ssODN library and forward strand patterns of the index PCR product. (**a**) Design of the ssODN library. Reverse was used in the enrichment and is shown here. The primer highlighted in blue contains a sequence, complementary to the Illumina sequencing primer, which allows skipping one PCR in the NGS preparation (see details in NGS part below). The ssODN library has a staggered design to ensure the diversity while sequencing the constant region. The resulting library consists of 4 ssODNs, which differ from each other in length by+1, +2, and +3 nucleotides (shown in green; see details in NGS part below). There is no substantial homo-dimerization possible between constant regions, which ensures that primarily the variable region will drive conformational changes in the complete aptamer sequences. Additional balancing was introduced to the design of the 5′ constant region to ensure the diversity during NGS (shown in red). (**b**) Patterns of the forward strands of the index PCR product. The 3′-end was attached to the surface of the flow cell. Arrow: beginning of the sequencing.

We used a random ssODNs library of molecules comprising a 35-nt variable region flanked by two fixed primer-binding sites (Fig. 6a). The library was synthesized by Integrated DNA Technologies (IDT, USA) as “ultramer” (high-fidelity version of the synthesis) and purified by standard desalting.

The design of the ssODN library, along with the Illumina HiSeq precision sequencing allowed us to sequence this library directly without diversity spike-in. GC content is equalized to 50% in the variable and constant regions. Before enrichment, each library was amplified with 5′-phosphorylated forward primer 5′-TCG TCG GCA GCG TCA-3′ (Tm: 62.6 °C), and 5′-biotinylated reverse primer 5′-CTA GCA TGA CTG CAG TAC GT-3′ (Tm: 61.1 °C). DNA amplification was performed on the 96-well Veriti thermal cycler (ABI). The final concentrations of reagents were 1X Q5 PCR reaction buffer (#M0493L, NEB), 0.2 mM dNTPs (#362271, Life Technologies), 1 μM of each anti-sense (5′ phosphorylated) and sense (5′ biotinylated) primer (IDT), 2 units of Q5 DNA polymerase (#M0493L, NEB), 0.01 pmol of pure library (or 33 μl of aptamer library sample recovered in PEG precipitation, see enrichment/profiling protocols below), and the final volume was brought to 100 μl with molecular grade H_2_O. Q5 DNA polymerase was chosen due to high fidelity and ability to work in a broad range of GC content, which is critical for handling aptamer libraries. The thermal cycling profile began with a 98 °C incubation for 30 s, followed by 10 cycles of 98 °C for 30 s, 60 °C for 30 s and 72 °C for 1 min, and final elongation at 72 °C for 5 min. The PCR product was purified using the NucleoSpin PCR clean-up kit (#740609, Macherey-Nagel) in NTI buffer according to the recommendation by the manufacturer. DNA was quantified with a QuBit ds BR/HS assay kit (#Q32853, Q32854, Life Technologies). The dsDNA library was mixed with Lambda Exonuclease (#M0262L, NEB) in the ratio 100 ng/5 units, 1X Lambda buffer, and the final volume was adjusted with molecular grade H_2_O. The digestion mix was incubated at 37 °C for 2 hrs. Subsequently, the enzyme was inactivated at 80 °C for 10 min. ssDNA was purified using NucleoSpin columns with NTC buffer (740654.1, Macherey-Nagel). Eluted ssDNA was quantified with the QuBit ssDNA assay kit (#Q10212, Life Technologies).

### Enrichment of aptamer library

As the initial step in developing ADAPT, an enriched library of randomized single-stranded DNA (ssDNA) sequences, called the profiling library L3, was generated from approximately 2 × 10^13^ different synthetic ssDNAs (Fig. 1d, library L0). In step 1, an aliquot of 10^11^ sequences of PCR-amplified L0 (Supplementary Data Fig. 1d) was incubated with pooled blood-plasma from 59 breast cancer patients with positive biopsies (Source A, Supplementary Table S3). In parallel, another aliquot of 10^11^ sequences was incubated with pooled blood-plasma from 30 patients with suspected breast cancer who proved negative on biopsy and 30 self-declared healthy women (Source B, (Supplementary Table S4). EVs were UC precipitated from both L0-samples (Fig. 1d, step 2). EV-associated oligodeoxynucleotides (ODNs) were recovered from the respective pellets. To drive the selection pressure towards enrichment of ODNs specifically associated with each sample cohort, a counter-selection step was carried out by incubation of each enriched library with plasma from the different cohorts (Fig. 1d, step 3), where the sequences contained in the EV pellets were discarded. In a second positive selection (Fig. 1d, step 4), the sequences contained in the respective supernatants (sn) from step 3 were mixed with plasma from another aliquot of each positive control sample-population, and EVs were again isolated. EV-associated ODNs were PCR amplified directly from the precipitate and converted to ssDNA as described above. Thus, recovered libraries are representing two single-round libraries called library L1 for positive biopsy patients, and library L2 for the control patients. In a final step, L1 and L2 were amplified by PCR, reverted to ssDNA, and mixed to yield library L3. This enrichment scheme was iterated two times more using L3 as the input to further reduce the complexity of the profiling library to approximately 10^6^ different sequences. The first iteration used UC for partitioning to increase the specificity for the EV fraction. The second two iterations used the same scheme (Fig. 1d), except for partitioning done by PEG-precipitation. Of note, a total of biopsy-positive (n = 59), biopsy-negative (n = 30), and self-declared normal (n = 30) were used in the first round of L3 enrichment, while only the cancer and non-cancer samples were used in the subsequent rounds.

#### Technical note on UC versus PEG EV purification from plasma samples

As we demonstrated in the Supplementary Figure 1b–e, the number of EV-associated proteins is similar in EV purifications done either with UC or PEG. However, the UC protocol takes 6–8 hours, while the PEG protocol requires 30 min at most. This technical advantage prompted the decision to switch from UC to PEG during enrichment and especially to employ PEG for massive screening of the naïve samples with trained libraries. Since the protein content (determined by MS) and size (determined by DLS; see below) was consistent between UC and PEG PPT with exosomes this enriched EV population is referred to as exosomes.

### Protocol for profiling clinical samples with enriched or synthetic libraries

Frozen plasma (1.0 ml) was treated with 50 μl of protease inhibitor (20X stock of protease inhibitor; from two tablets of *complete ULTRA MINI EDTA-free EASYpack* (5892791001, Roche) protease inhibitor in 1050 μl of H_2_O), and thawed. Cells/debris were removed by centrifugation (10000 × g, 20 min, 4 °C), and the supernatant was collected. To 1.0 ml of supernatant, 1.0 ml of 2X PBS (SH3025601, GE Healthcare) containing 6.0 mM MgCl_2_ was added and thoroughly mixed. Competitors were added to 3 aliquots of 400 μl for a final concentration of 0.01 mM dextran sulfate (APT-B006, Aptamer Sciences), 0.8 ng/μl salmon sperm DNA (15632-011, Life Technologies) and tRNA (AM7119, Life Technologies) in 1X PBS, 3.0 mM MgCl_2_ and thoroughly mixed. ssDNA aptamer probing library was added to a final concentration of 2.5 pg/μl with dilutions made in 1X PBS, 3.0 mM MgCl_2_, and incubated for 1 hr at ambient temperature with rotation. For partitioning, a 20% PEG8000 (P1458-50ML, Sigma-Aldrich) polymer solution in 1X PBS containing 3.0 mM MgCl_2_ was prepared and added to the sample to a final PEG concentration of 6%. The samples were inverted two times to mix, incubated for 15 min at 4 °C, and centrifuged (10,000 × g, 5 min, ambient temperature). The supernatant was removed, 1.0 ml of 1X PBS with 3.0 mM MgCl_2_ was added and the pellet was washed by gentle inversion. The buffer was removed; the pellet was resuspended in 100 μl H_2_O by shaking on the mixmate (Eppendorf, 900 rpm, 10 min, ambient temperature). Complete resuspension was confirmed by pipetting up and down, and the resuspended sample was used directly in the preparation for next generation sequencing (NGS).

### Next Generation Sequencing library preparation and HiSeq sequencing protocol

To prepare each library for sequencing we adopted Illumina’s two-step, tailed PCR approach (http://support.illumina.com/content/dam/illumina-marketing/documents/products/other/16s-metagenomics-faq-1270-2014-003.pdf). To skip the 1^st^ PCR we incorporated Illumina’s amplicon forward primer sequence into the constant region of the ssODN library (see highlighted in blue in Fig. 6a). Illumina also recommends using 4 forward primers in the 1^st^ PCR to allow sequencing of low diversity libraries. Since we are skipping the 1^st^ PCR, we incorporated staggered design directly into the library (see highlighted in green bases in Fig. 6a). The benefits of one-step NGS library preparation are: (i) high-throughput NGS sample preparation due to 5 hour shorter protocol; (ii) improved specificity of NGS data due to skipping one PCR step in the protocol and, accordingly, reduced number of mutants in the ssODN library; (iii) improved data quality and yield due to sequencing the starting point on the library variable region, since the variable region provides sufficient diversity in early sequencing cycles, which is critical for establishing clusters location and their density; (iv) lower error rate and shorter sequencing time due to the one read sequencing through the constant region and index, which allows unambiguous identification of ssODN sequences as well as de-multiplexing, without the need for separate sequencing of the index sequence.

#### One-step NGS prep protocol

Reversed strand library (0.01 pmol of pure library template; 1/2-1/3 of the PEG precipitated samples after particular enrichment round or profiling test) was amplified with 1 μM of index primers (IDT) (N7xx: CAA GCA GAA GAC GGC ATA CGG AT [i7] CTA GCA TGA CTGCAG TAC GT, and S5xx: AAT GAT ACG GCG ACC ACC GAG ATC TA AC [i5] TCG TCG GCA GCG TCA), 1X Q5 PCR reaction buffer (#M0493L, NEB), 0.2 mM dNTPs (#362271, Life Technologies), 2 units of Q5 DNA polymerase (#M0493L, NEB) and final volume was brought to 100 μl with molecular grade H_2_O. The thermal cycling profile began with a 98 °C incubation for 30 s, followed by 10 cycles (18 for post-binding sample) of 98 °C for 30 s, 60 °C for 30 s and 72 °C for 1 min, and final elongation at 72 °C for 5 min. Index PCR products were purified with AMPure beads (Beckman Coulter) according to Illumina standard protocol. Purified product was quantified with QuBit dsDNA kit (Q32854, Life Technologies), samples concentrations were normalized and then diluted to 14 nM before multiplexing.

#### HiSeq2500 sequencing set up for Rapid run mode (single read)

5 μl of each sample (2 nM) was mixed to make particular multiplex, then 5 μl of the pool was mixed with 5 μl of 0.2 M NaOH and incubated for 5 min at RT. This solution was further diluted to 7 pM with chilled HT1 buffer, denatured at 96 °C for 2 min, and snap-cooled in 3:1 ice:water bath for 5 min. Sequencing was performed on the HiSeq 2500 (Illumina) using cluster kit #GD-402-4001 TruSeq Rapid SR Cluster Kit - HS and sequencing kit #FC-402-4002 TruSeq Rapid SBS Kit (50 cycle, enough for 74 cycles) (Illumina). No diversity spike-in was used for sequencing of the starting and enriched libraries. NGS of the L2000 library required 25% spike-in of the non-enriched library due to the limited number of unique ssODNs.

*HCS version*: 2.2.58 (Nov 2014); *Library version*: 3.8.346; *Scanner*: RTA version 1.18.64; *Chemistry*: v2.0340.

#### Post-NGS data processing

After completion of sequencing, HiSeq Q score quality metrics were inspected to ensure the high accuracy of the base calling. Only runs with at least 85% of bases above Q30 (probability of correct base call is 99.9%) were considered. Runs with lower percentage were repeated.

HiSeq raw sequencing data were converted to the “.fastq” file according to the specification of bcl2fastq package from Illumina. The patterns of the correct constant region (highlighted in red in Fig. 6b), as well as the sequences of the index (i7) and anchoring adaptor (black) were used for alignment and de-multiplexing. Individual sample NGS output was further analysed by extraction of the sequences with the correct right constant region and generating an output file with the raw counts of the unique ssODN sequences. For a preliminary analysis of ssODN libraries we used: total output sequence number; total valid sequence number (aligned or mapped to the constant region of ssODN library); total number of unique ssODNs and at different read count cut-off; percentage of unique ssODNs sequence at different cut-off out of total number of unique ssODN; total raw counts from unique ssODNs at different cut-off; percentage of that total raw counts from unique ssODNs at different cut-off out of the total output for particular samples; average raw count per unique ssODNs sequence. The depth of the sequencing run was defined as the number of sequence tags, mapped to the constant region of the ssODN library. For comparison between different samples the normalized counts were used, which is reflecting the relative abundance of a particular ssODN sequence in the total NGS outcome for a particular sample. Detailed data mining is described in the Chapter “NGS data analysis” below.

### qPCR

Quantitative real-time PCR (qPCR) amplification was performed with regular amplification primers (see PCR protocol above) using SYBR Green Master Mix (VWR, #101414-286). The final concentrations of reagents in qPCR were 1X SYBR Green Mix, 1.0 μM of each forward and reverse primer (IDT), 5 μl of aptamer library probing sample, and volume was brought to 20 μl with molecular grade H_2_O. DNA standard was set in the range 5 × e^2^ to 5 × e^8^ copies. Each sample and standard was run in duplicate. qPCR was performed on the ViiA 7 Real-Time PCR System (Applied Biosystems). The thermal cycling profile began with a 95 °C incubation for 30 s, followed by 40 cycles of 95 °C for 10 s, 60 °C for 1 min. ViiA 7 software was used to generate a standard curve and to calculate the number of copies of the recovered ssODN library.

### Cell culture and exosome isolation

#### Cell culture

FBS (20% in DMEM) was depleted of bovine exosomes by centrifugation at 100,000 × g for 16 h at 4 °C. VCaP cells were cultured in DMEM supplemented with 10% depleted FBS, 2 mM L-glutamine, 1 U/mL penicillin, and 1 μg/mL streptomycin at 37 °C and 5% CO_2_. Exosomes were isolated from VCaP cell culture supernatant by sucrose density centrifugation. Briefly, supernatant was cleared of cells and cellular debris by sequential centrifugation at 400 × g for 10 min and 2000 × g for 20 min at 4 °C. Cleared supernatant was concentrated by centrifugal filtration (Centricon Plus-70, 100 kDa NMWL), layered on a 30% sucrose cushion and centrifuged at 100,000 × g for 75 min at 4 °C. Supernatant was removed and discarded without disrupting the cushion interface, which was then collected, diluted 6-fold with PBS and centrifuged at 100,000 × g for 70 min at 4 °C. The resulting exosome pellet was resuspended in PBS by pipetting and incubation overnight at 4 °C, then stored at −80 °C.

### Ultracentrifugation

Exosomes were isolated from human plasma by direct ultracentrifugation. Pooled plasma from normal female donors was diluted 1:1 with PBS then centrifuged at 2000 × g for 30 min at 4 °C to eliminate contaminating cells, followed by 12,000 × g for 45 min at 4 °C to clear any remaining platelets and cellular debris. Exosomes were pelleted by centrifugation at 120,000 × g for 2 h at 4 °C. Pellets were washed twice by resuspension in PBS and subsequent centrifugation at 120,000 × g for 70 min at 4 °C. The final exosome pellet was resuspended and stored as described above.

### Flow cytometry

Plasma exosomes were prepared by the direct ultracentrifuge method described above and diluted to 0.04 μg/μL in binding buffer (1X PBS plus 3 mM MgCl_2_). Exosomes (0.02 μg/μL) were blocked with 0.8 ng/μL salmon sperm DNA, 0.8 ng/μL tRNA, 0.01 mM dextran sulfate in binding buffer for 1 h at RT. Biotinylated aptamer libraries were incubated with equimolar streptavidin-phycoerythrin (SAPE, Invitrogen) in binding buffer for 30 minutes at RT. SAPE-labeled aptamers were then diluted to 12 nM with blocked exosomes (1 μg) and incubated for 1 h at RT. In some cases, subsequent to aptamer binding, exosomes were stained with 32 μM CFDA SE and 5 μM DiD (Invitrogen) for 15 min at 37 °C. To assess aptamer binding to exosomes, PE fluorescence intensity associated with exosome events was measured by flow cytometry (A50-Micro, Apogee Flow Systems).

### Aptamer library pull-down of target proteins from plasma

#### SBED library conjugation, binding and crosslinking

The L2 library enriched against normal female plasma and L0 (as control) were conjugated with sulfo-SBED (Thermo Scientific) as described^36^ with minor modifications. SBED library functionalization was tested by performing the ADAPT™ assay with SBED vs DMSO mock conjugated control C6-amine library and sequenced on a HiSeq 2500^TM^ (Illumina) as described above. The aptamer precipitation was then done with forty-eight ADAPT™ reactions incubated for 1 h with end-over-end rotation at room temp with a 5 ng input of SBED conjugated library per 200 μL of plasma (pre-spun to remove cellular debris at 10,000 × g for 20 min, 4 °C) in 1X PBS, 3 mM MgCl_2_, 0.01 mM dextran sulfate, 40 ng/μl salmon sperm DNA and 40 ng/μl yeast tRNA, and cOmplete ULTRA Mini EDTA-free^TM^ protease inhibitors (Roche) equivalent to ~240 ng library and 9.6 mls plasma. A duplicate set of 48 reactions was prepared with the DMSO control C6-amine library. Aptamer library-protein complexes were precipitated with incubation in 6% PEG8000 for 15 min at 4 °C then centrifuged at 10,000 × g for 5 min. Pellets were washed with 1 ml 1X PBS, 3 mM MgCl_2_ by gentle inversion to remove unbound aptamers. The washed pellets were resuspended in 100 μL of water and subjected to photo-cross-linking at 365 nm with a hand-held 3UV (254NM/302NM/365NM) lamp, 115 volts (Thermo Scientific) for 10 min on ice with 1–2 cm between the 96-well plate and lamp.

#### Aptamer precipitation and target identification

Cross-linked reactions were subsequently pooled (~4.8 ml) per library or 4.8 ml of 1X PBS (AP bead only control) and incubated with 10 μL of Prepared Dynabeads^®^ MyOne™ Streptavidin C1 (10 mg/ml)(Life Technologies) (pre-washed with 1X PBS, 0.01% Triton X-100) shaking for 1 h at room temp. Beads were transferred to an eppendorf tube and lysed for 20 min with lysis buffer (50 mM Tris-HCl, 10 mM MgCl_2_, 200 mM NaCl, 0.5% Triton X-100, 5% glycerol, pH 7.5) on ice, washed 3 times with wash buffer 1 (10 mM Tris-HCl, 1 mM EDTA, 2 M NaCl, 1% Triton X-100), followed by 2 times with wash buffer 2 (10 mM Tris-HCl, 1 mM EDTA, 2 M NaCl, 0.01% Triton X-100) as described^2^. Cross-linked proteins were eluted by boiling 15 min in 1X LDS sample buffer with reducing agent added (Life Technologies) and loaded on a 4–12% SDS-PAGE gradient gel (Life Technologies). Proteins and DNA were detected with double staining with Imperial Blue Protein Stain (Thermo Scientific) followed by Prot-SIL2^TM^ silver stain kit (Sigma) according to manufacturers instructions in order to enhance sensitivity and reduce background. Band slices were cut out and sent to TGen Center for Proteomics (Phoenix, AZ) for in-gel digestion and detection on a Thermo LTQ Orbitrap Velos. Bands common to both SBED-conjugated and control libraries were subtracted and those unique to the SBED-conjugated library were analysed for GO enrichment analysis (http://geneontology.org/page/go-enrichment-analysis).

### Target identification

Prepared Dynabeads^®^ MyOne™ were immobilized with 100 μL H1, H11, L4, or L15 @ 0.15 mg/ml or No Oligo control in 50 mM HEPES, pH 8.3, 3 mM MgCl_2_, 0.5 M NaCl, 0.2 mM EDTA, 0.1% Tween-20 for 30 min at room temperature. Unbound ssODNs were removed by washing with the immobilization buffer and beads were equilibrated with 1X PBS, 3 mM MgCl_2_. PEG precipitation of plasma from healthy donors was performed as described in section “**Protocol for profiling clinical samples with enriched or synthetic libraries”,** except bead-immobilized ssODNs were used. Prepared beads were incubated for 30 min at room temperature with 100 μL of a 10-fold dilution of PEG-precipitated plasma from healthy donors, subjected to end for end rotation for 10 min at room temperature with 0.8 ng/μL of sheared salmon sperm DNA, 0.8 ng/μL yeast tRNA, and 0.01 mM dextran sulphate. Beads were then lysed in 20 mM HEPES-KOH, 3 mM MgCl_2_, 200 mM NaCl, 0.5% Triton X-100, 0.1% Tween-20, pH 7.4, and subsequently washed with 2 additional times with lysis buffer and three times with 20 mM HEPES-KOH, 3 mM MgCl_2_, pH 7.4 and eluted in 0.3% TFA, 6 M Urea. 4X Pierce™ LDS Sample Buffer (Life Technologies) was added to 1X final concentration with 1X NuPAGE^®^ Sample Reducing Agent (Life Technologies) and loaded onto a NuPAGE™ Novex™ 4–12% gradient gel (Life Technologies). Proteins were separated for either 45 min (for Fig. 5) or 10 min (barely run in to remove PEG), and detected by staining gels with Imperial Blue Protein Stain (Thermo Scientific). A band slice from the 10 min separation encompassing the entire range >10 kDa to the stack were cut out. Peptides were extracted from sliced bands with a Thermo In-Gel Tryptic digestion kit ™ according to manufacturer’s instructions and cleaned up with Pierce™ C18 Tips. 50% of the sample was injected onto a PepMap RSLC column (C18, 2 μm, 100 Å, 75 μm × 25 cm) with a Thermo Scientific™ Acclaim™ PepMap™ RSLC Nano Trap Column, and eluted at a flow rate of 300 nL/min with a 35 min gradient (0.1% HCOOH, 1.6% CH_3_CN to 0.1% HCOOH, 40% CH_3_CN) with a Dionex UltiMate 3000 RSLCnano system, and detected using a Thermo Q-Exactive HF Hybrid Quadrupole-Orbitrap Mass Spectrometer (QE HF). A blank run was injected between each sample and eluted with the same gradient. The QE HF was operated in data-dependent mode with the top 10 most intense ions determined from the high resolution (60,000) MS survey scan (scan range: m/z 375–1800; AGC 3 × e^6^) followed by HCD acquisition (resolution 15,000; scan range m/z 200–2000, AGC 1 × e^5^). Dynamic exclusion was applied with repeat count and exclusion duration of 1 s and 5 s, respectively. Protein identifications were performed using Proteome Discoverer version 2.1 and Sequest HT Search as the database search engine on Homo sapiens (SwissProt TaxID = 9606), Streptomyces avidinii (SwissProt TaxID = 1895) v. 2015-09-16 exported from Swissprot. and custom cRAP v. 2012.01.01 (http://www.thegpm.org/crap/) databases (for common contaminants) filtered by high confidence.

### Transmission Electron Microscopy (TEM)

EM and immuno-EM analysis of vesicles were performed as described previously^37^ with minor modifications. Specifically, the vesicle pellet was resuspended in 1X PBS and deposited onto a Formvar coated slide of copper mesh EM grids (FF300-Cu-50, Electron Microscopy Sciences). The vesicle-coated grids were washed, stained with 1% UO_2_(CH_3_COO)_2_ and then viewed for transmission EM (TEM) using a Philips CM12; camera: Gatan model 791. For the immuno-gold labelling with antibodies, blocked grids were transferred to a drop of the antibody in 0.1 M NaPO_4_ pH 7.2/150 mM NaCl/1% BSA/0.01% Tween20 and incubated for 30 min. The grids were then washed, incubated with gold-labeled secondary antibody in 1X PBS/1% BSA/0.01% Tween20 for 20 min. After washing with the same buffer and deionized water, the grids were stained with 0.5% UO_2_(CH_3_COO)_2_ and then imaged by TEM.

### Dynamic Light Scattering

Exosomes samples were prepared by PEG-precipitation of blood plasma as described above, with titration of PEG 1%, 2%, 4%, 5%, 6%, 8%. Vesicle size distribution was measured with DynaPro Plate Reader II (Wyatt Technology Corporation, Santa Barbara, CA) using equal volumes of each sample, in three replicates, having 5 acquisitions of 5 seconds each at 25 °C. PBS buffer and UC-isolated exosomes were used as control.

### Western Blot

PEG precipitation of plasma sample was performed according to the probing protocol described above using 4, 5, and 6 final % of PEG without the addition of competitor or aptamer library. After final re-suspension step sample protein content was measured by nanodrop. Samples (50 μg of recovered plasma sample) were separated on 4–12% Bis Tris protein gel (NP0322BOX, Invitrogen). Control for Ab performance was 0.05 μg exosome sample from the VCaP cell line, isolated by UC. Proteins were transferred to a nitrocellulose membrane (IB301001, Invitrogen) by iBlot standard procedure. Primary antibody probing was performed with 1 μg/mL mouse *anti*-CD9 ab (MAB1880, R&D Systems) followed by 1:10,000 diluted goat *anti*-mouse HRP secondary antibody (115-035-062, Jackson Immuno Research) and final development of signal by 5 min incubation with vendor recommended concentration of chemiluminescent substrate (34096, Thermo). Image of blot was captured by imager (PXi, Syngene) following 10 s exposure.

### Filter retention assay

#### ssODN-C1Q interaction analysis

Radioactively labelled ssODNs were obtained by *in vitro* kinase reactions using T4 PNK and γ-[^32^P]-ATP (PerkinElmer, Waltham, USA). Reaction mixtures were purified using NucleoSpin PCR and Gel Clean-up kits (Machery and Nagel, Düren, Germany) according to the manufacturers instruction. Trace amounts of ^32^P-labelled ssODNs were incubated with increasing C1Q concentrations in binding buffer (PBS, pH7.4, supplemented with 6 mM Mg^2+^ and 10% C1Q-depleted serum) and incubated at 37 °C for 30 min. Afterwards, the mixture was passed through nitrocellulose membranes (0.45 μM, Schleicher and Schuell Biosciences GmbH, Dassel, Germany) and washed with 800 μl binding buffer w/o serum. The amount of radioactively labelled ssODNs bound to protein was detected with a FLA-3000 (Fujifilm, Tokyo, Japan) and quantified using AIDA image software (raytest GmbH, Straubenhardt, Germany).

### Enzyme-Linked Oligonucleotide Assay (ELONA)

Purified C1Q Protein (CompTech, Tyler, USA) was directly coated overnight at 4 °C onto a high binding 96 well plate (Corning Costar, Corning, USA). Plates were then blocked with PBS, pH7.4, supplemented with 3 mM MgCl_2_, 0.1 mg/ml sheared salmon sperm DNA, 0.1 mg/ml yeast tRNA, 0.08 mg/ml dextran sulfate and 3% BSA) for 1 h at 22 °C. The wells were then washed 3 times each with wash buffers 1 (20 mM HEPES-KOH, pH 7.4, 3 mM MgCl_2_, 200 mM NaCl) and wash buffer 2 (20 mM HEPES-KOH, pH 7.4, 3 mM MgCl_2_) respectively. Biotinylated ssODN was pre-incubated with streptavidin-poly HRP (Pierce, Waltham, USA) for 15 min at 22 °C. This mixture was added to the wells and incubated for 1 h at 22 °C. The wells were washed 3 times each with wash buffer 1 and 2. The amount of ssODN bound to protein was detected with a HRP substrate with absorbance at 450 nm (Biotek, Vinooski, USA).

### NGS data analysis

See preliminary data analysis above in post-NGS processing part.

The number of copies of a particular ssODN sequence was normalized by dividing this number by the total number of counts and multiplying it by the global mean.

#### ANOVA Permutation test

Number of ANOVA F test significant aptamers was tested by 1000 permutations of the sample labels. A one-way ANOVA model was built for each aptamer using normalized count. Aptamers were considered significant when their F test p values were less than 0.05.

#### Random Forest Models

Random forest models were realized using the random Forest function from the random Forest R package. Specifically, the model contained 1000 decision trees. The number of random features was determined by the square root of number of features input into the model (default). The prediction probability was computed based on out-of-bag (OOB) samples. Area under the ROC curve was calculated to evaluate the model performance. The OOB AUC was also tested by 1000 permutations of the sample labels. The permutated labels were then used to compute the corresponding AUCs.

## Additional Information

**How to cite this article:** Domenyuk, V. *et al*. Plasma Exosome Profiling of Cancer Patients by a Next Generation Systems Biology Approach. *Sci. Rep.* **7**, 42741; doi: 10.1038/srep42741 (2017).

**Publisher's note:** Springer Nature remains neutral with regard to jurisdictional claims in published maps and institutional affiliations.

## Supplementary Material

Supplementary Information

Supplementary Table S1

Supplementary Table S2

Supplementary Table S3

Supplementary Table S4

Supplementary Table S5

Supplementary Table S6

Supplementary Table S7

Supplementary Table S8

Supplementary Table S9

Supplementary Table S10

Supplementary Table S11

Supplementary Table S12

Supplementary Table S13

Supplementary Table S14

Supplementary Table S15
