# Supplementary material for: Plasma Exosome Profiling of Cancer Patients by a Next Generation Systems Biology Approach
Source: Sci Rep. 2017 Feb 20;7:42741. doi: 10.1038/srep42741 (PMC5316983; doi:10.1038/srep42741)
Supplement: Supplementary Information [file srep42741-s1.pdf]

Supplementary Information

Plasma Exosome Profiling of Cancer Patients by a Next Generation Systems Biology Approach

Valeriy Domenyuk, Zhenyu Zhong, Adam Stark, Nianqing Xiao, Heather A. O'Neill, Xixi Wei, Jie Wang, Teresa T. Tinder, Sonal Tonapi, Janet Duncan, Tassilo Hornung, Andrew Hunter, Mark R. Miglarese, Joachim Schorr, David Halbert, John Quackenbush, George Poste, Donald A. Berry, Günter Mayer*, Michael Famulok*, and David Spetzler*

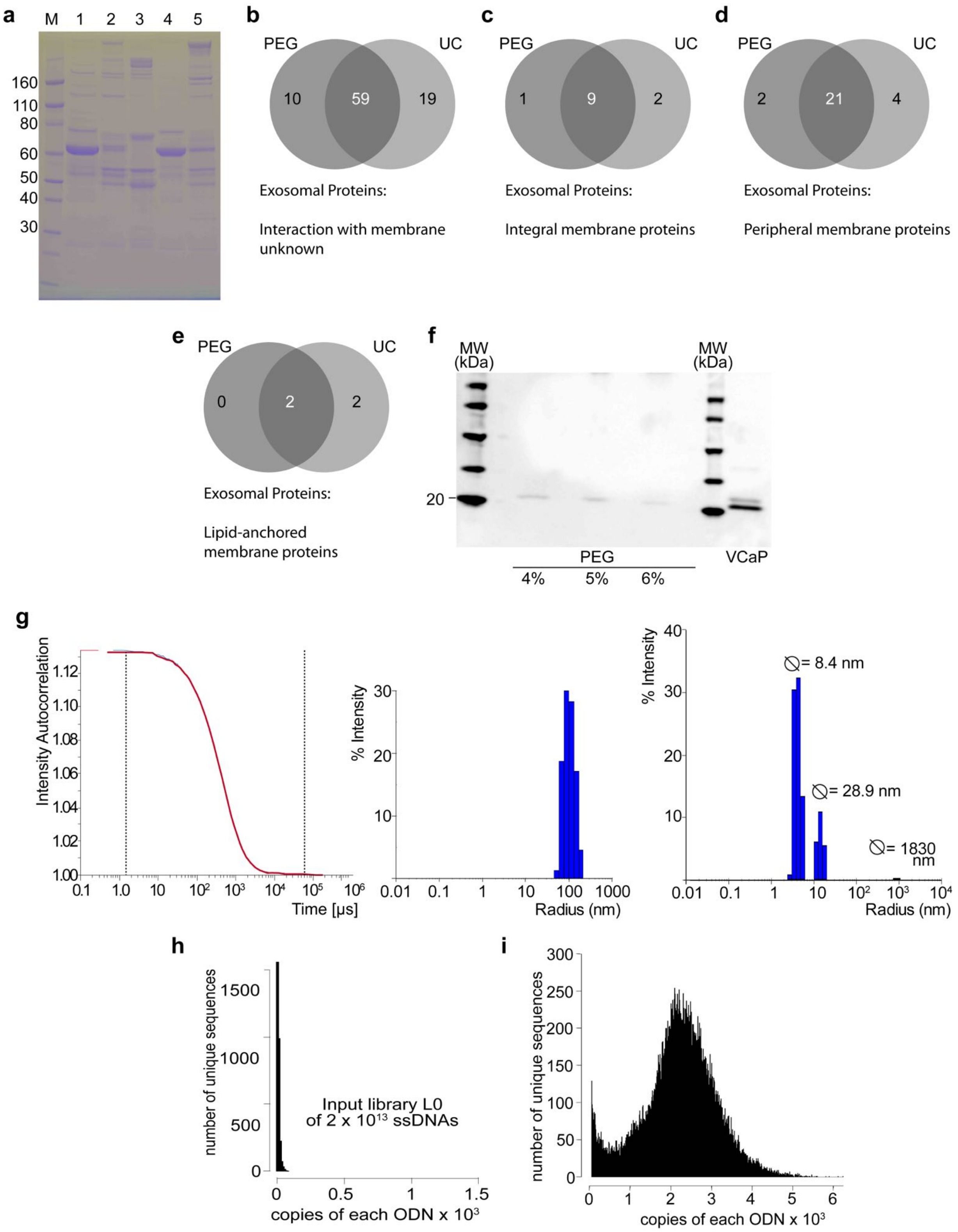

Supplementary Figure 1. PEG precipitated plasma characterization and usage for ODNs enrichment

(a) Plasma fractionation by PEG-precipitation compared to Ultracentrifugation (UC). Imperial blue-stained gel shows the difference between the proteins profiles. Lane 1: neat plasma; lane

2: plasma pellet PEG-precipitated; Lane 3: plasma pellet UC-precipitated; Lane 4: over night depleted supernatant; Lane 5: overnight UC depleted supernatant followed by PEG-precipitation.

(**b**) Proteins identified in PPT and UC and their relation to exosomes: interaction with membrane is not known. The data are from: Human Membrane Protein Analysis System (HMPAS) http://fcode.kaist.ac.kr/hmpas/page.jsp?curPage=keysearch.

(**c**) Proteins identified in PPT and UC and their relation to exosomes: integral membrane proteins. The data are from: human membrane protein analysis system.

(**d**) Proteins identified in PPT and UC and their relation to exosomes: peripheral membrane proteins. The data are from: human membrane protein analysis system.

(**e**) Proteins identified in PPT and UC and their relation to exosomes: lipid-anchored membrane proteins. The data are from: human membrane protein analysis system.

(**f**) Western blot analysis of exosomes, isolated with 4%, 5% and 6% PEG8000 (50 μg loaded), showed the target band when probed with anti-CD9 antibody (expected MW 24 kDa). VCaP: Control experiment using exosomes isolated from the cell line VCaP (10 ng loaded) by ultra-centrifugation.

(**g)** Dynamic light scattering study of PEG- and UC-precipitated particles. Left panel: Signal decay curve confirming the robustness of the DLS data shown in Figure 1b. Middle Panel: DLS of breast cancer plasma exosomes, purified by UC. Right panel: "No plasma" control DLS of human serum albumin, precipitated with 6% PEG8000.

(h) Input library L0 characterization. Histogram of unique sequences number versus read count distribution in the input library.

(**i**) Profiling library L3 characterization. Histogram of unique sequences number versus read count distribution in the enriched library. For proper representation of enrichment of unique sequences, original NGS data L3 (d) were consolidated, so that any offspring mutated sequences will be removed.

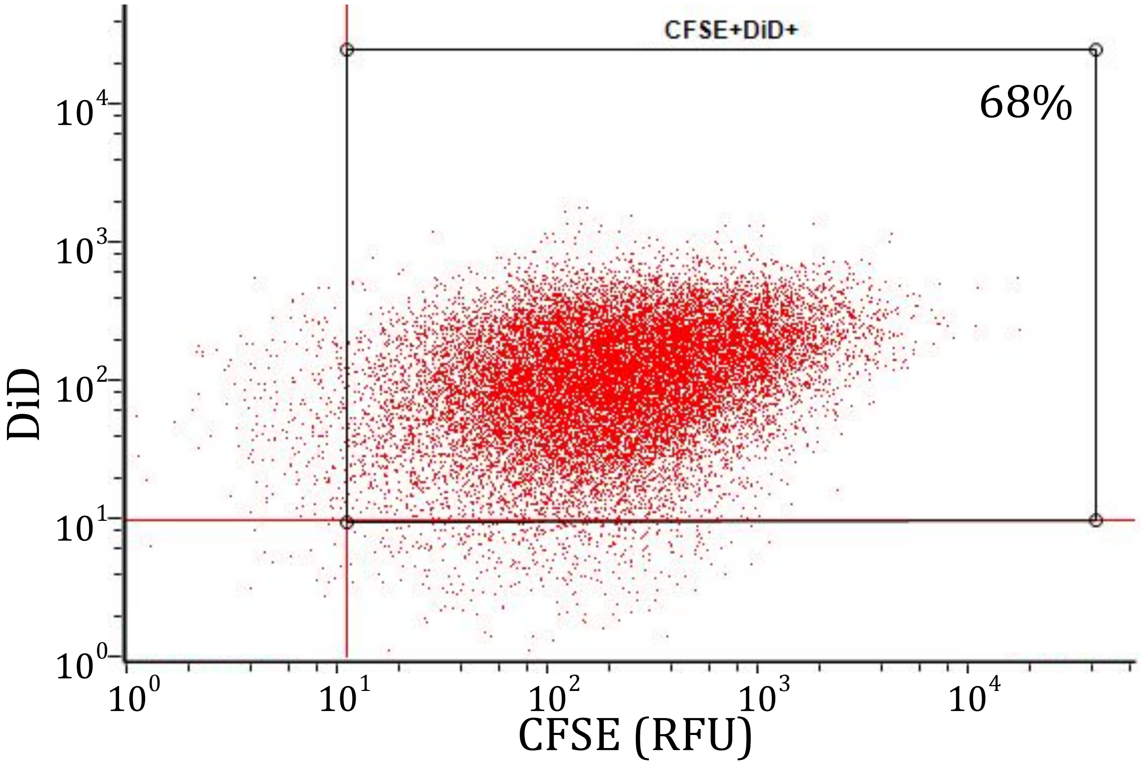

Supplementary Figure 2. Initial gating strategy (representative example of triplicate samples) of the flow cytometry experiment in Figure 2a, showing light scatter events double-positive for both CFSE and DiD staining (68%), which were further evaluated for SA-PE staining.

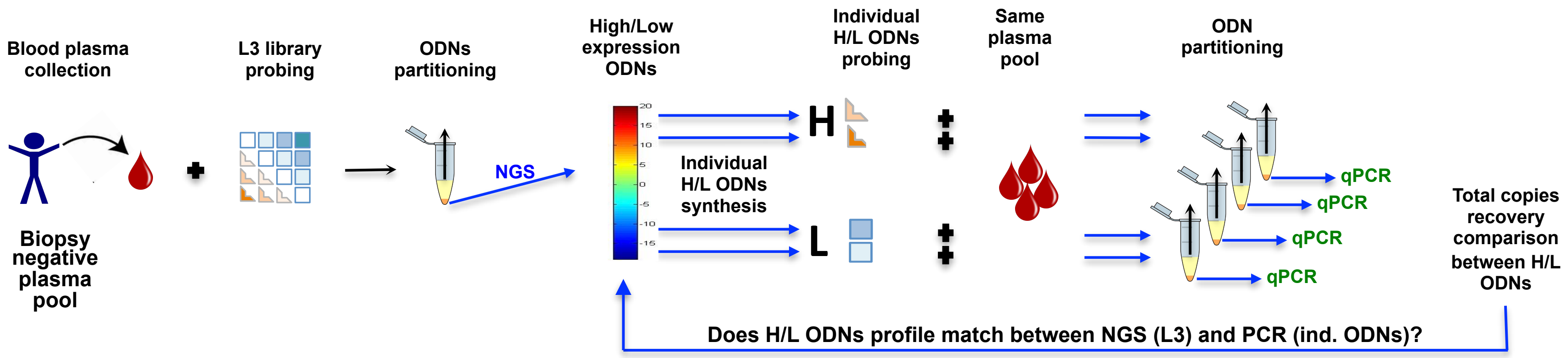

Supplementary Figure 3. Workflow for comparison of the expression profiles of individual ssODNs in binding blood plasma (pool of breast biopsy negative donors) as part of library (L3) or individually. Plasma pool was probed with the L3 library according to the standard ADAPT workflow (3 replicates per binding reaction), followed by NGS analysis. Representative sequences with relatively high (H) and low (L) expression were selected based on normalized counts of individual ssODNs, and resynthesized. H ssODNs have ≥5-fold higher expression than L ssODNs. Validation of individual H/L ssODNs was performed using the same probing protocol and the same plasma samples (pooled) utilizing orthogonal assay (qPCR) as a readout (3 binding replicates per ssODN). The rationale for this test was to verify the binding of individual ODN that were isolated from the library and had equal concentration, using an alternative to the NGS readout assay. Most of the ssODNs, selected from the L3 library as H showed higher total recovery in qPCR as well, compared to the control L ssODNs, which were underrepresented in L3 (most likely PCR mutants).

In addition, to verify the impact of the mutation on binding properties we included several mutants (1-2 bases change) of the higher counts H ssODNs. Indeed, 2 mutations H3→H7 and H2→H12 led to a significant increase in binding, shown by qPCR. Another mutant of H2 (H8) had similar recovery in binding.

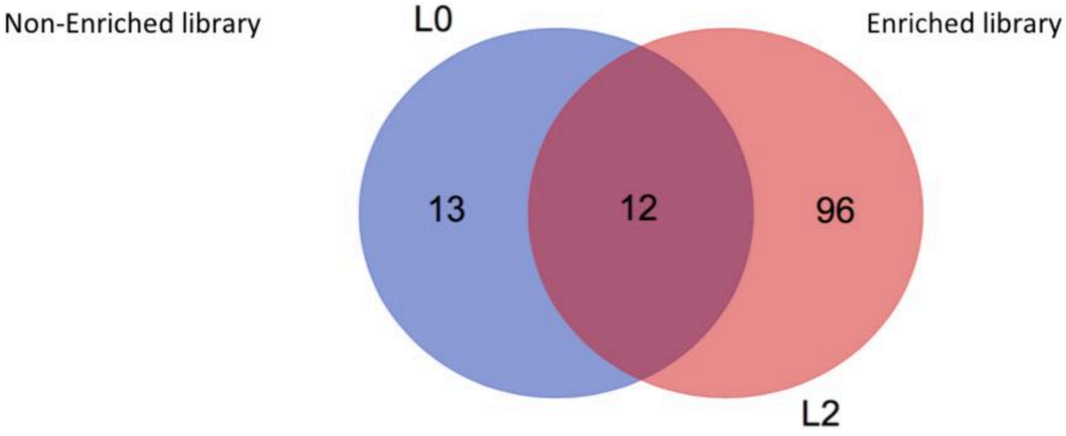

Non-exosomal proteins:	1	0	2
Integral membrane proteins:	0	1	5
Peripheral membrane proteins:	2	5	28
Lipid-anchored membrane proteins:	0	0	3
Interaction with membrane is not defined:	10	6	58

Supplementary Figure 4. Origin of protein targets of ADAPT identified with ssODNs contained in L0 and in L2 as defined in Supplementary Table 7 using the following databases:
Vesiclepedia (http://www.microvesicles.org);
Compartments (http://compartments.jensenlab.org/Search);
Human Membrane Protein Analysis System (HMPAS) (http://fcode.kaist.ac.kr/hmpas).

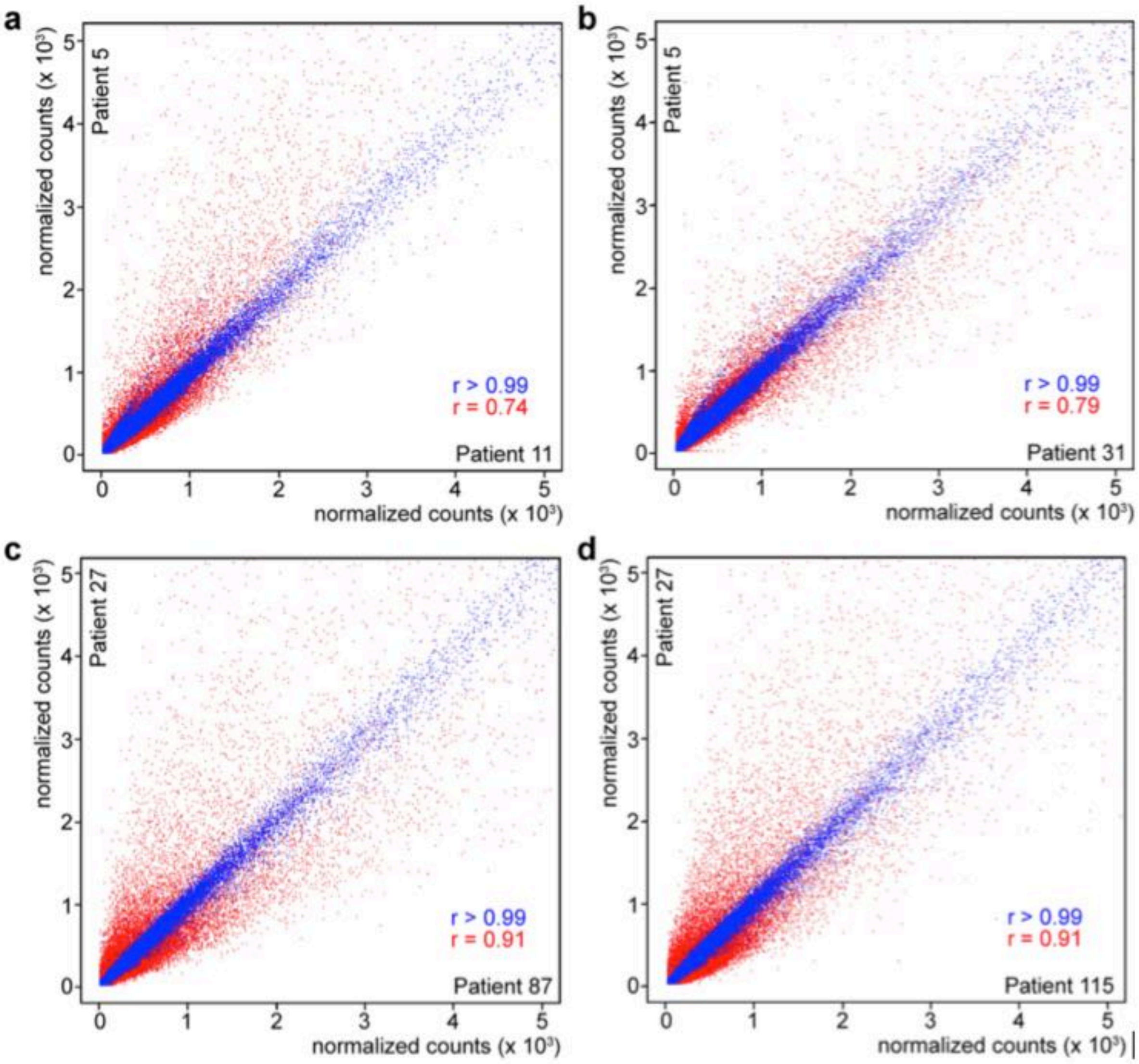

Supplementary Figure 5. Additional examples to Fig. 3b, showing the correlation between technical replicates and biological samples for L3. Scatter plots show the distribution of normalized counts of aptamers recovered from ADAPT with the plasma aliquots of the same patient (blue dots, $r > 0.99$) or plasma samples from different patients (red dots, $r = 0.74$-0.91). Every individual dot represents a unique sequence with the count of that sequence corresponding to representation in different samples or technical replicates. (**a**) Inter sample: patient 5 vs. patient 11 (red dots), intra sample: patient 5 (blue dots), (**b**) Inter sample: patient 5 vs. patient 31 (red dots), intra sample: patient 5 (blue dots), (**c**) Inter sample: patient 27 vs. patient 87 (red dots), intra sample: patient 27 (blue dots), (**d**) Inter sample: patient 27 vs. patient 115 (red dots), intra sample: patient 27 (blue dots).

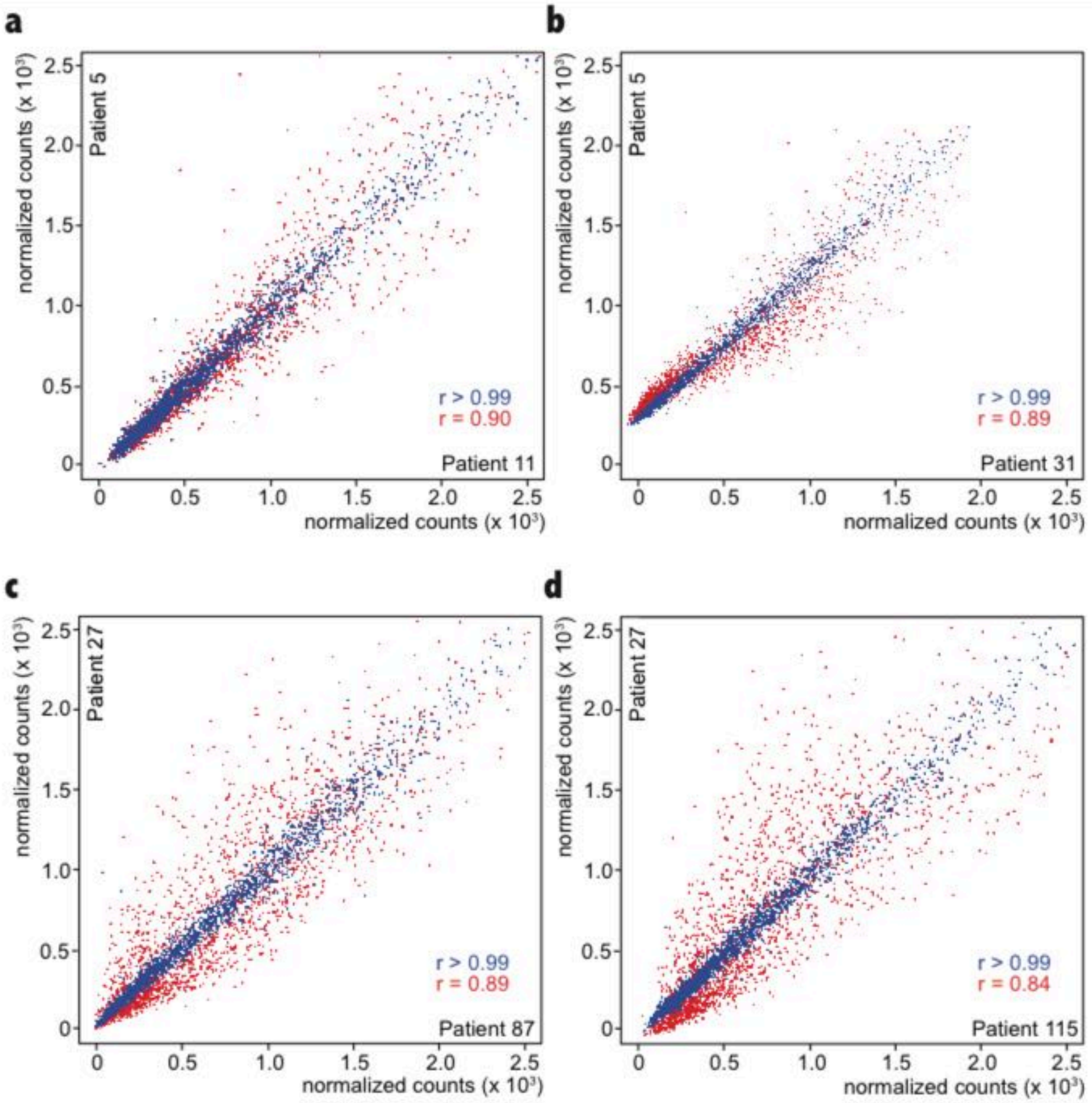

Supplementary Figure 6. Additional examples to Fig. 3c, showing the correlation between technical replicates and biological samples for L2000. Scatter plots show the distribution of normalized counts of aptamers recovered from ADAPT with the plasma aliquots of the same patient (blue dots, $r > 0.99$) or plasma samples from different patients (red dots, $r = 0.84$-0.90). Every individual dot represents a unique sequence with the count of that sequence corresponding to representation in different samples or technical replicates. (**a**) Inter sample: patient 5 vs. patient 11 (red dots), intra sample: patient 11 (blue dots), (**b**) Inter sample: patient 5 vs. patient 31 (red dots), intra sample: patient 31 (blue dots), (**c**) Inter sample: patient 27 vs. patient 87 (red dots), intra sample: patient 87 (blue dots), (**d**) Inter sample: patient 27 vs. patient 115 (red dots), intra sample: patient 115 (blue dots).

Supplementary Table Captions

Table S1. List of common and unique proteins, identified with mass spectrometry analysis in plasma after UC or PPT, with specification of their association with exosomes.

Table S2. Sequences of 2000 ODNs, selected from 4 different enrichments and used for profiling of 500 clinical samples.

Table S3. Demographic table of the training set for the 59 breast cancer patients with positive biopsy.

Table S4. Demographic tables of the training set for 60 individuals with breast cancer negative biopsy.

Table S5. Next generation sequencing data and QC metrics

Table S6. Sequences of 23 ODN's from L3 library with high (H) and low (L) recovery in binding individual plasma samples.

Table S7. List of proteins identified with mass spectrometry analysis in plasma, affinity purified with either L0 (non-enriched) or L2 (enriched) library. Controls are proteins, identified in plasma after UC and PPT. Note, plasma pool for controls is different from the one used to generate data for Table S1.

Table S8. Library L0 enriched proteins, analyzed by GO cellular component enrichment.

Table S9. Library L2 enriched proteins, analyzed by GO cellular component enrichment.

Table S10. Library L2 enriched proteins, analyzed by GO molecular function enrichment.

Table S11. Library L2 enriched proteins, analyzed by GO biological process enrichment.

Table S12. Demographic table of the patients' cohorts, used in 4 enrichments, specified in Table S10. Note, samples from the Tables S1 and S2 are included in this table.

Table S13. Demographic table of the individual patients, used in screening of enriched libraries (Table S10) to select 2000 ODNs (Table S10).

Table S14. Demographic table of 206 breast cancer biopsy positive patients used for profiling with L2000 library

Table S15. Demographic table of 177 breast cancer biopsy negative patients used for profiling with L2000 library.
